# Featuring the application of biochemistry in dental practice through a self-directed assignment: A descriptive study

**DOI:** 10.1371/journal.pone.0347026

**Published:** 2026-04-10

**Authors:** Nazlee Sharmin, Ava K. Chow

**Affiliations:** Mike Petryk School of Dentistry, Faculty of Medicine & Dentistry, College of Health Sciences, University of Alberta, Canada; Mae Fah Luang University School of Anti Aging and Regenerative Medicine, THAILAND

## Abstract

**Background:**

With advancements in the fields of treatment, diagnosis, and genomics, knowledge of cellular biochemistry is becoming increasingly crucial for oral health professionals. However, dental students often feel demotivated to study foundational-level biochemistry because they are overwhelmed by the volume and complexity of the material and fail to recognize its relevance to dental practice. To address this, we developed a focused, self-directed assignment for 1^st^-year Dental Hygiene (DH) students to enhance their understanding of protein structure and underscore the application of this knowledge in dental practice.

**Methods:**

The assignment was developed in quiz-style using Google Forms and integrated into a foundational-level biochemistry course as a supplementary activity. The activity included follow-up questions asking students about their perceived accomplishments and confidence in understanding protein structure and function. A descriptive study was conducted to explore students’ perceptions of the assignment and its impact on their understanding of protein structure and function. Students’ anonymous responses to the follow-up questions and their performance on the assignment were analyzed for the study. Descriptive statistics were calculated from the data using Microsoft Excel.

**Results:**

Although the assignment was not mandatory, 86% of the class (n = 25) completed it, achieving an average score of 6.04 out of 10. Sixty percent of participants (n = 15) reported improved knowledge of protein structure-function relationships, and 48% (n = 12) indicated that completing the assignment helped them understand how a simple change in protein sequence can lead to significant changes in protein structure and function.

**Conclusion:**

While the assignment included a limited number of follow-up questions, which restricted our ability to explore student perceptions in detail, it demonstrates the potential of a well-designed, interactive, case-based assignment to stimulate student motivation by highlighting the relevance and application of biochemistry content in the future dental profession.

## Introduction

Dentistry and dental hygiene are established branches of human health sciences that treat and manage diseases of the mouth, teeth, and related tissues, significantly impacting our wellbeing [1]. As diagnostic and treatment methodologies continue to evolve, it has become increasingly crucial for oral health professionals to have a deep understanding of the molecular mechanisms underlying the oral biosystem [2]. A strong body of evidence shows that genetic mutations can alter protein structure, dynamics, stability, and expression, leading to disrupted binding, impaired protein interactions, loss of functional domains, and protein degradation [3–10]. These biochemical changes contribute to developmental anomalies of the teeth and craniofacial structures [3 6]. Therefore, a solid grasp of mutations, and their biochemical effects on protein structure and function is vital for oral health professionals to fully understand the etiology and pathogenicity of various congenital dental and oral diseases [2,11].

Besides the well-recognized value of biochemistry in dental education, students often fail to identify the relevance of this discipline to their dental practice and thus become overwhelmed and demotivated by the volume and complexity of the material [2,12]. In a study conducted among 125 dental students, Ramalingam et al. reported that 69% of participants disliked biochemistry, as they found the content to be of little relevance to clinical practice. In the same study, while students acknowledged the importance of biochemistry in diagnosing and predicting oral diseases, a significant number also admitted to not fully understanding the principles behind biochemistry lab experiments [2]. In a similar study, Youhanna et al. also reported demotivation of dental students towards biochemistry and their desire to recognize the relevance of this subject to dentistry [12]. Traditionally, biochemistry is taught through a combination of didactic lectures and laboratory exercises. At the Mike Petryk School of Dentistry in Canada, a 2.0-credit introductory biochemistry course is offered to 1st-year Dental Hygiene (DH) students, covering protein structure and function, enzyme kinetics, lipids, carbohydrates, metabolism, and nucleic acids over 30 hours of didactic lectures. This content-heavy course does not allow sufficient class time to discuss the application of foundational biochemistry knowledge in dentistry and dental hygiene practice, necessitating an alternative approach to help students recognize its relevance and importance in these fields.

Self-directed learning, with some guidance, is becoming a popular approach in medical education to deliver vast amounts of knowledge that cannot be fully conveyed in lecture classes [13,14]. Self-directed learning is essential for medical and dental students to become lifelong learners, where they can self-identify their learning needs, formulate learning objectives, and evaluate the learning process [15,16]. However, one of the challenges with self-directed learning with large and complex content is to make students focus and understand the key concepts [14]. Guidance in the form of instruction [13], questions [14], supporting lecture [17] or discussion [16] has been shown to improve students learning outcome compared to self-directed learning without any guidance. It is also recommended that first-year health professional students receive training before engaging in self-directed learning, as they may not yet have fully developed the necessary skills for it [17,18].

Considering the importance of biochemistry, we developed a focused, self-directed assignment for Dental Hygiene (DH) students to improve their understanding of protein structure and to highlight the application of this knowledge in dental practice. A study was conducted to answer the following research questions:

1. What learning benefits do students perceive from completing the self-directed assignment?
2. To what extent does the assignment affect students’ perceived understanding of protein structure and function? Addressing these questions will help clarify how students value this type of focused, self-directed assignment as a learning resource. We hypothesize that the self-directed assignment will positively influence students’ perceived understanding of protein structure and function and help them recognize the application of biochemistry in dental practice.

## Materials and methods

### Study design

A descriptive, cross-sectional study design was chosen for this research, which takes a systematic approach to describe a population, situation, or phenomenon at a single point in time without identifying the underlying cause [29,30]. This type of study aims to answer what, where, when, and how questions about an intervention or phenomenon, but not the ‘why’. This type of study design does not aim to manipulate variables or establish cause-and-effect relationships. Therefore, instead of comparing a treatment group to a control group, descriptive studies aim to provide a comprehensive picture through methods like surveys, case studies, and observational studies [29,30].

### Assignment design and implementation

The assignment revolves around multiple clinical cases, guiding students to analyze and interpret protein sequences, structures, and functions. The assignment, along with its case descriptions, guided activities, and critical thinking questions, was embedded in a Google Form in the format of a quiz. Once students submitted the assignment, they could see their scores, the correct answers, and a brief explanation supporting each correct answer. Some key features of the assignment include:

i. Incorporation of clinical cases and protein structures

**Fig 1:**
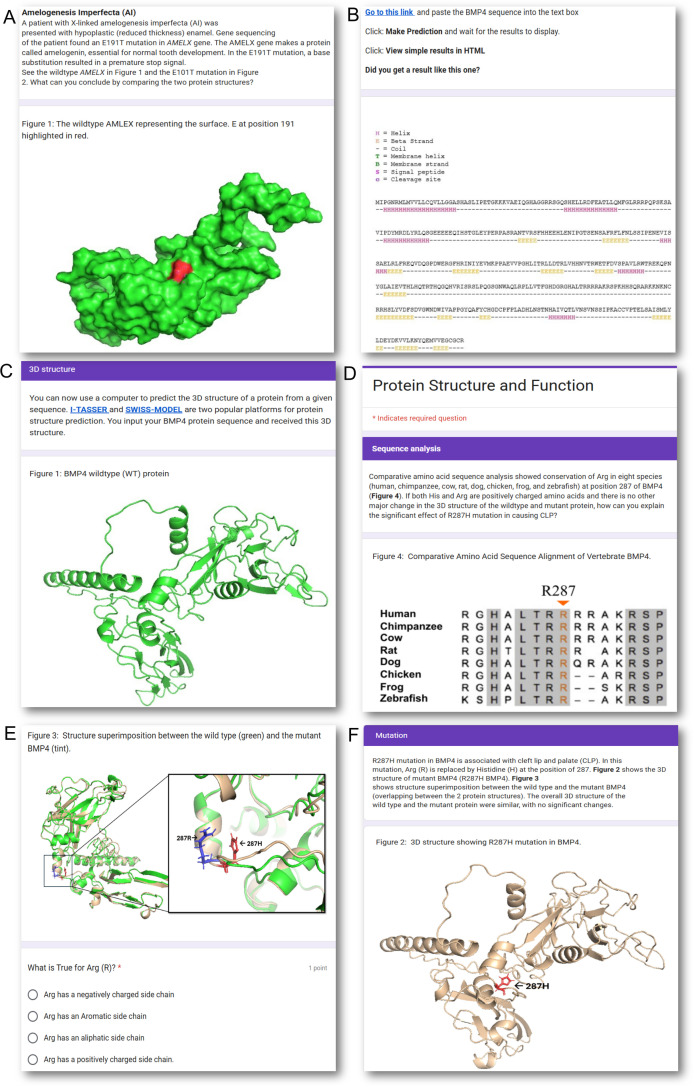
Screenshot from various parts of the biochemistry assignment. The assignment was made using Google Forms. The 3D protein models were generated using I-TASSER [25].

The assignment includes two clinical scenarios related to cleft lip and palate (CLP) and Amelogenesis Imperfecta (AI). CLP has a diverse genetic background, involving mutations in several genes [19–23]. The assignment featured a mutation in the bone morphogenetic protein (BMP) gene for CLP [22] and a substitution mutation in the AMELX gene for AI [23]. At the beginning of the assignment, students were provided with a protein sequence from a patient with CLP. They were then guided to acquire the secondary and tertiary structure of the protein, identify the mutation in the patient, and interpret the possible effect of the mutant protein through sequence and 3D structure analysis. The second part of the assignment includes a case of AI and a substitution mutation in AMELX gene. The sequence and mutant data used in this assignment were obtained from previously published literature [22,23] (Fig 1A). As of December 2025, no structural data were available in PDB [24] for the human BMP4 or Amelogenin (encoded by the AMELX gene). Therefore, *in silico* models of the wild-type and mutant forms of BMP4 and Amelogenin were generated using I-TASSER [25].

ii. Exposure to bioinformatic tools

Students were encouraged to use several freely accessible bioinformatics tools as part of this assignment, which included JPred4, a server for protein secondary structure prediction [26], as well as I-TASSER [25] and SWISS-MODEL [27], two widely used platforms for predicting three-dimensional protein structures. Structural comparisons between the wild-type and mutant proteins were performed using PyMOL [28], and the resulting visualizations and data were provided to the students. Based on these results, students were required to answer a series of related questions (Fig 1B, 1C).

iii. Critical thinking questions

Along with regular knowledge-base questions, a series of critical-thinking questions was included in the assessment, aiming to solidify and challenge students’ concepts of protein structure and function (Fig 1D). Upon submission, students received detailed feedback with an explanation of why a certain answer choice was correct.

iv. Review and apply knowledge acquired in the classroom

Part of the assessment was aimed at helping students review the knowledge of protein structure that they learned in the didactic lecture and apply it in a different context (Fig 1E).

v. Freedom to explore knowledge beyond the classroom

Part of the assessment was aimed at offering students the option and freedom to explore knowledge beyond what is learned in the classroom (Fig 1F). The assignment was posted in the Learning Management System (LMS) as a supplementary activity. Questions embedded in the assessment provided immediate feedback, helping students to self-assess their understanding.

The learning outcomes of the assignment, as expected by the instructor, included:

- Recognize the relevance of the knowledge of biochemistry (protein structure) in oral health.
- Reinforce the knowledge of the primary, secondary, and tertiary structures of protein (previously discussed in the didactic lecture).
- Identify the impact of mutations on the structure of a protein.
- Recognize the impact of a mutation on the function of a protein.
- Comprehend the relationship between protein structure and function.
- Become familiar with the availability of tools for sequence analysis.

**Fig 2:**
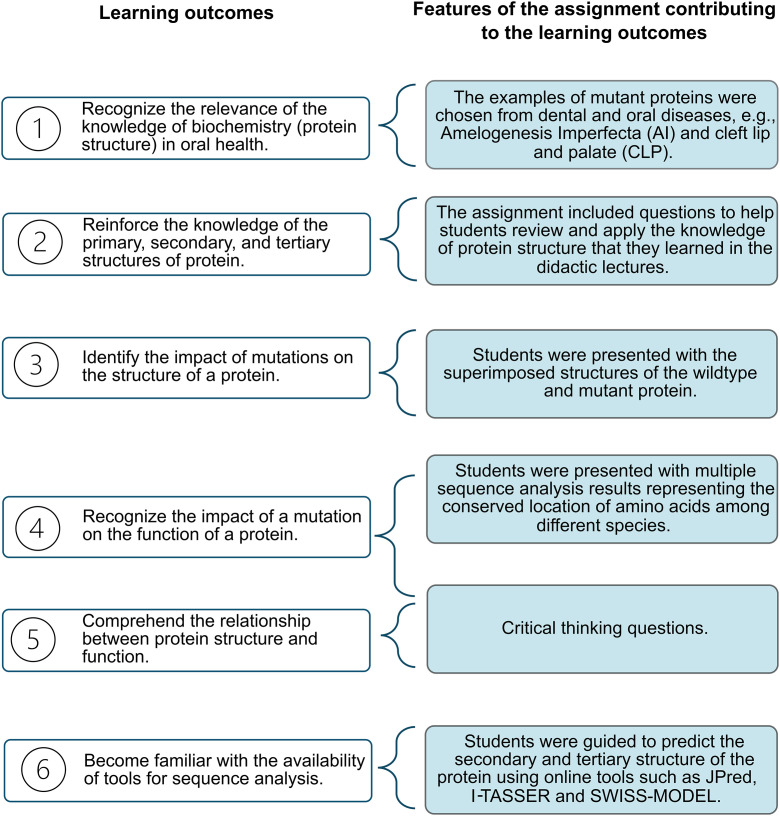
Features of the assignment contributing to the instructor-defined learning outcomes.

The features of the assignment that contributed to the instructor-defined learning outcomes are outlined in Fig 2.

### Study population

1^st^-year DH students, who enrolled in the foundational-level Biochemistry course (OBIOL 203) in the Winter semester of 2025, and submitted the assignment, were the participants of the study. A census sampling was employed, i.e., all students who submitted the assignment (n = 25) were included in the study, there were no exclusion criteria. The assignment, created in Google Forms, was incorporated into the OBIOL 203 course as a supplementary activity. The link for the assignment was posted in the LMS and was not graded.

### Ethics statement

The study was conducted between January 20 and April 10, 2025, in Canada. The Research Ethics Board of the University of Alberta has approved this study (ID: Pro00148848). The data were anonymized before analysis. The waiver of participant consent was approved by the ethics committee, as the data were anonymized, the assignment was not for grading, and this study has no impact on course grading.

### Data collection and analysis

The assignment did not collect any student-specific information. The cumulative performance of the class on various questions of the assignment was analyzed to assess the students’ level of understanding of the topic. The assignment included follow-up questions that inquired about students’ perceived accomplishments and confidence in understanding protein structure and function after completing the assignment:

- On a scale of 1–5, how confident are you about understanding protein structure and function?
- Check all that apply to you: By completing the assignment, _______
  a) I have improved my understanding of protein structure.
  b) I have improved my knowledge of structure-function relationships for proteins.
  c) I have understood how a simple change in protein sequence can lead to a significant change in protein structure and function.
  d) I found no benefit.
  e) Other:

The follow-up questions and scores are included in S1 Table. All the questions were author-developed and was not formally validated. Students’ anonymous responses to the follow-up questions were also analyzed for the study. No demographic data was collected. As the assignment was not graded and was a supplementary activity, students could complete it throughout the course. Data was downloaded after the course ended. Descriptive statistics were calculated from the data using Microsoft Excel.

## Results

### Participation and performance of the DH students in the supplementary assignment

**Fig 3:**
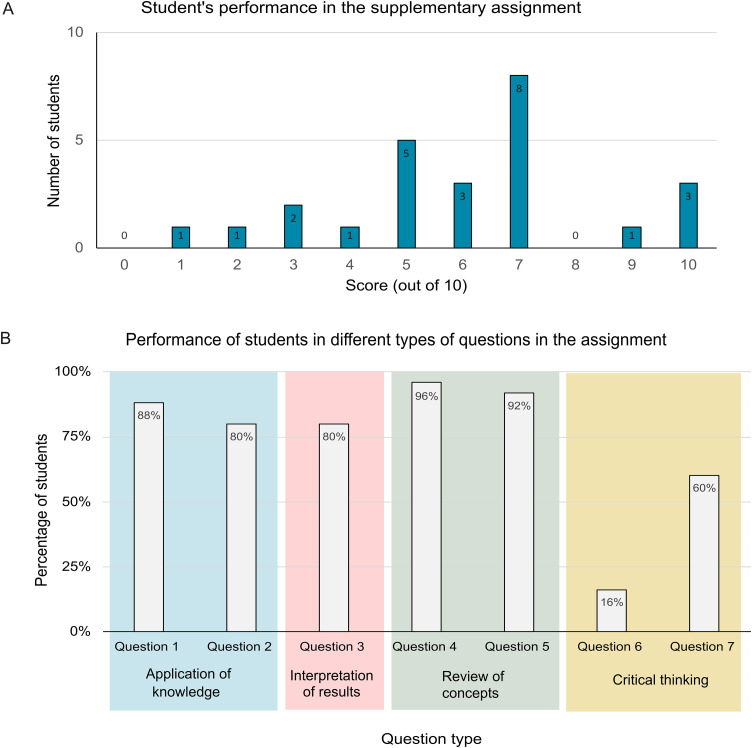
Students’ performance in the assignment. **(A)** The performance of the class in the supplementary assignment. The assignment was designed in Google Forms in the format of a quiz. Marks were calculated out of 10. **(B)** Performance of students in different types of questions in the assignment.

Although the assignment was supplementary to the regular lecture and was not counted towards the course grade, 86% of the class (n = 25) completed it, achieving an average score of 6.04 out of 10 (Fig 3A). The students performed best on the review of concept-type questions, followed by those requiring application of their theoretical knowledge. They showed the lowest performance on the critical thinking questions (Fig 3B).

### Students’ perceived outcome of the assignment

**Fig 4:**
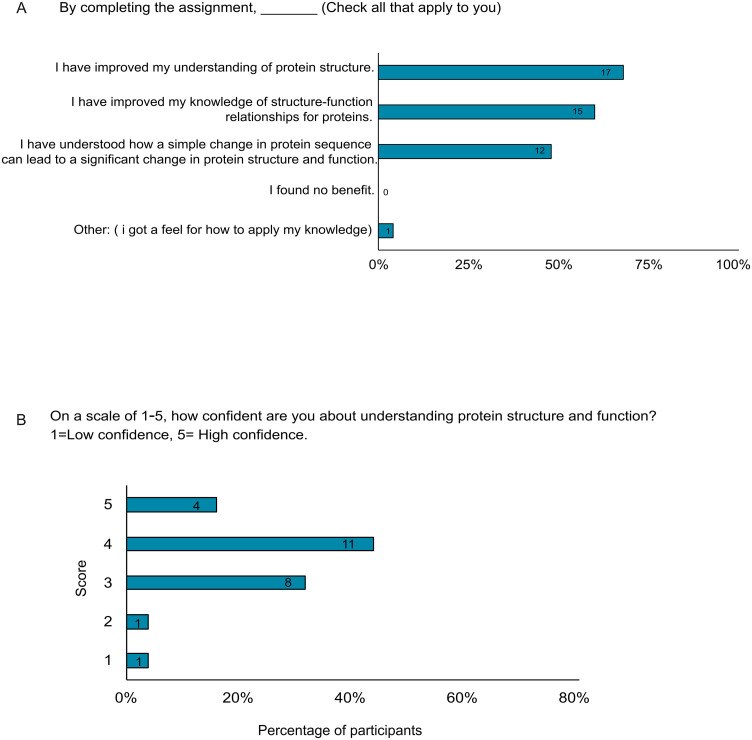
Students’ perception and confidence. **(A)** Students’ perceived outcomes of the biochemistry assignment. **(B)** Students’ self-reported confidence score after completing the assignment. Students were asked to rate their confidence in understanding protein structure and function on a scale of 1 to 5.

The majority (68%, n = 17) of the students who completed the assignment indicated that upon completing the assignment, they improved their understanding of protein structure. Sixty percent (n = 15) reported having improved their knowledge of protein structure-function relationships, and 48% (n = 12) implied that by doing the assignment, they had understood how a simple change in protein sequence can lead to a significant change in protein structure and function. Additionally, 4% (n = 1) of the students mentioned getting a feel for how to apply the knowledge learned in the classroom (Fig 4A).

### Students’ perceptions of the impact of the assignment on their understanding of protein structure and function

**Fig 5:**
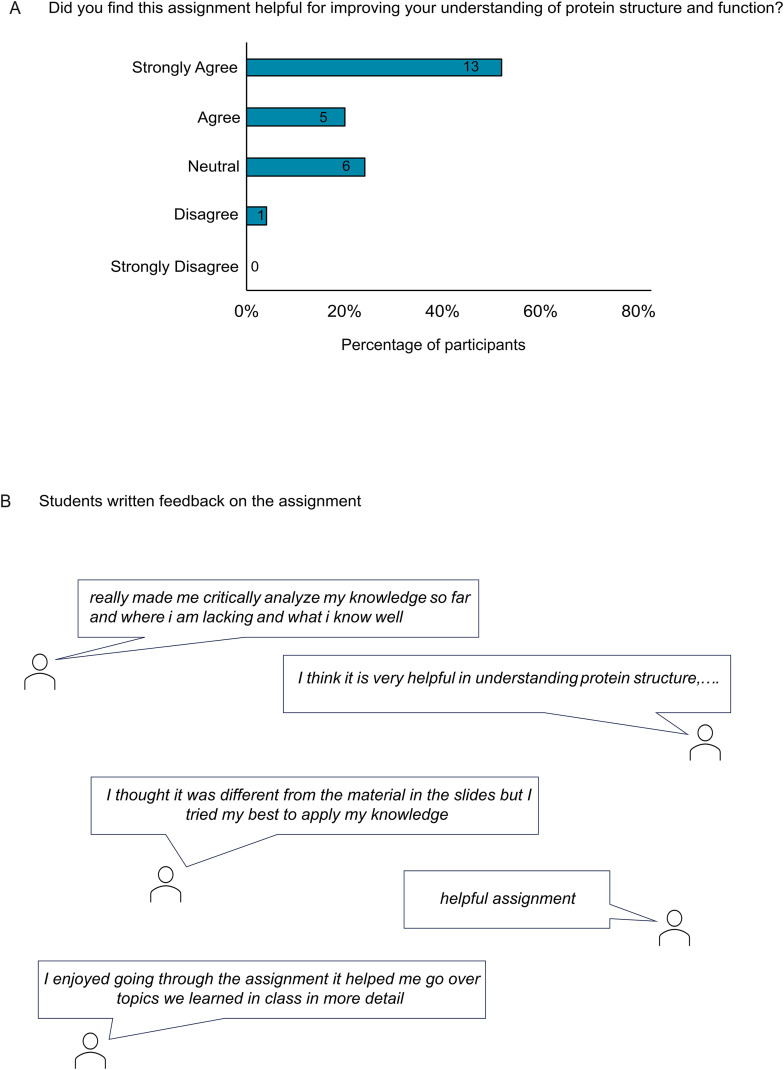
Students’ perceived impact of the assignment and written comments. **(A)** Students’ perceptions of the impact of the assignment on their understanding of protein structure and function. **(B)** Students’ comments on open-ended questions.

When students were asked to rate their confidence in understanding protein structure and function on a scale of 1–5, 16% (n = 4) of the students who completed the assignment rated their confidence as 5, 44% (n = 11) as 4, and 32% (n = 8) as 3 (Fig 4B). Over half of the students (52%, n = 13) who completed the assignment strongly agreed, and another 20% (n = 5) agreed that the assignment helped improve their understanding of protein structure and function. Four percent (n = 1), however, disagreed, and 24% (n = 6) remained neutral (Fig 5A). Students’ comments to open-ended questions also showed their positive attitude toward this activity (Fig 5B) (S1 Table).

## Discussion

Knowledge of biochemistry is essential for oral health professionals. However, many students become demotivated by the complexity of the content and fail to identify the application of this knowledge in their professional fields [2,12]. Foundational-level biochemistry courses are often designed to focus on the fundamental concepts of biomolecules and metabolic reactions, with little emphasis on their applications in clinical practice, which is a key element for motivating and engaging health professional students. We designed and implemented a simulated case-based assignment to help students recognize the potential application of their didactic biochemistry knowledge in the oral health profession.

The assignment was partly self-directed in nature and was implemented as an optional activity for students, supplementary to the didactic biochemistry lecture. The DH program at the University of Alberta is highly competitive, recruiting students of diverse ages and educational backgrounds. The assignment was made self-paced, non-grading, and optional, allowing students with different learning levels to engage with the material and benefit as needed. First-year DH students had not previously been introduced to sequence analysis tools. Instructions and suggestions were embedded to guide students on how to use the provided protein sequence and which key features to focus on in protein structure comparison. The follow-up questions included after the assignment were very limited and did not explore students’ opinions on various aspects of the assignment. However, in a similar study involving self-directed biochemistry activities with first-year MBBS students, Thota et al. reported that 87% of the participants found their sessions helpful for identifying strengths and weaknesses on the topic, and 93% agreed that the facilitator’s guidance helped them utilize the available resources effectively during the self-directed activity [17].

In the assignment, most students scored between 5 and 7, indicating that many students had an average understanding of protein structure-function relationships. The distribution of questions showed strong performance from students on questions that reviewed knowledge from the didactic lectures. Critical thinking, an essential component of effective problem-solving [31], tends to develop progressively as dental hygiene (DH) students advance through their curriculum [32]. The lower scores on critical-thinking questions may be attributed to the early academic stage of the DH students enrolled in the biochemistry course. In the same study, Partido et al. reported no significant correlation between DH students’ critical thinking abilities and overall academic performance [32]. Therefore, in our study, the first few years of DH students who performed well on critical-thinking items may not necessarily represent the highest-performing students in the class. An assignment like this one can also allow the instructor to formatively assess students’ understanding and help prepare instructional plans.

Students perceived learning outcomes of the assignment were well-aligned with many of those identified by the instructor. Students were asked to answer what they had achieved after completing the assignment. In addition to selecting from a list of predefined options, they were also given the opportunity to provide open-ended responses through an “Other” option. The results revealed that besides improving the understanding of protein structure and function, this assignment also helped students recognize the relevance of the biochemistry knowledge they learn in the classroom. Representing the relevance of a subject is the key to motivating and engaging adult learners [33]. A qualitative study on how medical students perceive the relevance of biomedical science knowledge showed that students deemed a topic “relevant” when it was directly applicable to patient care, such as diagnosis, management, safety, and education [34]. Consistent with these findings, our study demonstrated that students were able to identify practical applications of their knowledge after completing the case-based assignment, underscoring its value in bridging classroom learning with clinical relevance.

One of the key reasons students were found to be demotivated in biochemistry courses is the sheer volume and complexity of the material [2,12]. Traditional teaching methods often encourage rote memorization of structural and chemical data related to biomolecules, increasing students’ cognitive load. As a partial response to this challenge, the assignment aimed to introduce students to some existing bioinformatic tools for sequence and structural data analysis and visualization. This approach may help undergraduate students realize that essential biochemical data can be accessed, analyzed, and understood using digital tools, offering relief from the pressure to memorize vast amounts of information.

Completing the assignment served as an active learning experience for the students. According to Bonwell et al., active learning involves “instructional activities involving students in doing things and thinking about what they are doing” [35]. Over the course of the assessment, students engaged in various forms of active learning, including inquiry-based exploration, sequence analysis, evidence gathering, knowledge integration, and result interpretation. These tasks required critical thinking and hands-on engagement with the material. DH students fall within the category of emerging adults, and according to adult learning theories, such learners are intrinsically motivated, relevance-oriented, and learn best when they actively engage with the material [36,37]. The positive feedback expressed in both student responses and written reflections further supports the effectiveness of this active learning approach and aligns well with established principles of adult education.

### Limitations

We acknowledge several limitations of our study. The assignment included a limited number of follow-up questions, limiting our ability to explore students’ perception in detail. The data is collected from one cohort of DH students, no demographic data were collected. We also recognize that our findings are based on students’ self-reported data, which can be influenced by the responder’s capacity to interpret questions. The results rely on perception-based outcomes rather than objective measures of learning. No baseline data, pre-intervention assessment, control or comparison group were included in the study; therefore, it is possible that the observed outcomes can be a result of natural learning progression or other confounding factors. No pilot study was conducted. Since the questions were part of students’ assignments, the results may be affected by social desirability bias; participants might have been inclined to give responses they believed were more acceptable or expected. Future research should investigate students’ perceptions of various aspects of the assignment, including both quantitative and qualitative data.

## Conclusion and future plans

A strong knowledge and solid understanding of biochemistry is essential for any health professional. A thoughtful, interactive, case-based assignment can serve as a potential tool to stimulate student engagement and motivation by highlighting the relevance and application of biochemistry content in their future profession.

Future research can focus on incorporating pre- and post-knowledge assessments, including control groups, and involving larger study populations. Qualitative methods, such as focus groups, could also provide deeper insights into students’ experiences with the assignment. Additionally, variations in students’ performance and perceptions of the assignment’s benefits could be examined across demographic factors such as gender and age. Finally, investigating the long-term impact of such assignments would be valuable; for example, by interviewing students in later years of the program to explore whether attitudes toward biochemistry and its relevance to dental practice differ between those who completed the assignment and those who did not.

## Supporting information

S1 Table Students’ anonymous responses to the follow-up questions. (PDF)
