# Supplementary material for: Featuring the application of biochemistry in dental practice through a self-directed assignment: A descriptive study
Source: PLoS One. 2026 Apr 10;21(4):e0347026. doi: 10.1371/journal.pone.0347026 (PMC13068214; doi:10.1371/journal.pone.0347026)
Supplement: S1 Table — (PDF) [file pone.0347026.s001.pdf]

S1 Table **Students' anonymous responses to the follow-up questions**

Serial Number	Check all that apply to you:<br>By completing the assignment, _______	On a scale of 1-5, how confident are you about understanding protein structure and function? 1=Low confidence, 5= High confidence.	Did you find this assignment helpful for improving your understanding of protein structure and function?	Please provide your feedback on this assignment.<br>Submit the assignment to view your score and review your performance.
1	I have improved my understanding of protein structure., I have improved my knowledge of structure-function relationships for proteins., I have understood how a simple change in protein sequence can lead to a significant change in protein structure and function.	3	5	I think it is very helpful in understanding protein structure, however there are some question errors (eg. one of the options had "option 2" in the answer). Otherwise, I really liked this!
2	I have improved my understanding of protein structure.	3	3	n/a
3	I have improved my understanding of protein structure., I have improved my knowledge of structure-function relationships for proteins., I have understood how a simple change in protein sequence can lead to a significant change in protein structure and function.	4	5	I enjoyed going through the assignment it helped me go over topics we learned in class in more detail
4	I have improved my understanding of protein structure., I have improved my knowledge of structure-function relationships for proteins., I have understood how a simple change in protein sequence can lead to a significant change in protein structure and function.	3	2	I thought it was different from the material in the slides but I tried my best to apply my knowledge
5	I have improved my knowledge of structure-function relationships for proteins.	3	3	na
6	n	3	3	n
7	I have improved my understanding of protein structure., I have improved my knowledge of structure-function relationships for proteins., I have understood how a simple change in protein sequence can lead to a significant change in protein structure and function.	4	5	
8	I have understood how a simple change in protein sequence can lead to a significant change in protein structure and function.	4	5	good

9	I have improved my understanding of protein structure., I have improved my knowledge of structure-function relationships for proteins., I have understood how a simple change in protein sequence can lead to a significant change in protein structure and function.	3	4	
10	I have improved my knowledge of structure-function relationships for proteins.	4	3	n/a
11	I have improved my understanding of protein structure., I have improved my knowledge of structure-function relationships for proteins., I have understood how a simple change in protein sequence can lead to a significant change in protein structure and function.	4	5	good
12	I have improved my understanding of protein structure.	4	5	N/A
13	I have improved my knowledge of structure-function relationships for proteins.	4	4	N/A
14	I have improved my understanding of protein structure.	2	3	na
15	I have improved my understanding of protein structure.	5	5	na
16	I have improved my understanding of protein structure.	5	5	na
17	I have improved my knowledge of structure-function relationships for proteins.	4	4	NA
18	I have improved my understanding of protein structure., I have improved my knowledge of structure-function relationships for proteins., I have understood how a simple change in protein sequence can lead to a significant change in protein structure and function., i got a feel for how to apply my knowledge	4	5	really made me critically analyze my knowledge so far and where i am lacking and what i know well
19	I have improved my understanding of protein structure., I have improved my knowledge of structure-function relationships for proteins., I have understood how a simple change in protein sequence can lead to a significant change in protein structure and function.	3	4	Na

20	I have improved my understanding of protein structure., I have improved my knowledge of structure-function relationships for proteins., I have understood how a simple change in protein sequence can lead to a significant change in protein structure and function.	4	5	helpful assignment
21	I have improved my understanding of protein structure.	4	5	
22	I have improved my understanding of protein structure.	5	5	
23	I have improved my understanding of protein structure.	5	5	na
24	I have improved my knowledge of structure-function relationships for proteins., I have understood how a simple change in protein sequence can lead to a significant change in protein structure and function.,	1	3	n
25	I have improved my knowledge of structure-function relationships for proteins., I have understood how a simple change in protein sequence can lead to a significant change in protein structure and function.	3	4	no feedback
